# Impact of wheat straw incorporation and fertilizer reduction on peanut yield and soil functions

**DOI:** 10.3389/fpls.2026.1828860

**Published:** 2026-05-29

**Authors:** Yanyan Suo, Xiang Zhang, Liang Li, Xianzong Si, Fengdan Xu, Xingxiang Wang, Peijun Cheng, Qian Li, Meng Yan

**Affiliations:** 1 Institute of Plant Nutrition and Resource Environment, Henan Academy of Agricultural Sciences, Henan Key Laboratory of Agriculture Resources and Environment, Zhengzhou, China; 2 State Key Laboratory of Soil & Sustainable Agriculture, Institute of Soil Science, Chinese Academy of Sciences, Nanjing, China; 3 University of Chinese Academy of Sciences, Beijing, China

**Keywords:** *Arachis hypogaea* L., extracellular enzyme activity, fertilizer reduction, rhizosphere microbiome, soil aggregates, straw incorporation

## Abstract

**Introduction:**

Balancing crop productivity with sustainable soil management is a critical challenge in modern agriculture.

**Methods:**

We conducted a three-year randomized complete block field experiment (2022–2024) to evaluate the integrated effects of wheat straw incorporation regimes and fertilizer reduction on the plant-soil-microbe nexus in peanut (*Arachis hypogaea* L.) production. Six treatments were compared: no straw return (CT), conventional straw incorporation (SI), deep straw incorporation with a decomposition accelerator (SD), deep incorporation with accelerator and a 25% fertilizer reduction (SDR), surface mulching (SM), and SM with a 25% fertilizer reduction (SMR).

**Results:**

Relative to CT, deep straw incorporation (SD and SDR) significantly increased the three-year mean peanut yield by 12–25%. Notably, the SDR regime maintained final yields, plant nutrient uptake, and soil aggregate stability statistically equivalent to the fully fertilized SD treatment (*P* > 0.05), successfully substituting for a 25% reduction in mineral inputs. Mechanistically, deep straw incorporation actively reshaped the rhizosphere microenvironment. Deep straw incorporation actively reshaped the rhizosphere microenvironment, enriching beneficial functional microbial taxa including *Bacillus*, *Sphingomonas*, and *Trichoderma*, which subsequently elevated the activities of carbon, nitrogen, and phosphorus-cycling extracellular hydrolases and oxidative enzymes by 1.4- to 2.6-fold. This microbially mediated acceleration of nutrient cycling enhanced soil organic carbon, microbial biomass carbon, and available nutrient pools, thereby improving soil nutrient availability and peanut production capacity.

**Discussion:**

Our findings demonstrate that deep straw incorporation with targeted microbial decomposition accelerators, combined with a 25% reduction in mineral fertilizer, provides a practical, low-input strategy to sustain high peanut yields, optimize root-zone functions, and advance climate-smart agricultural systems.

## 1 Introduction

Straw incorporation is a promising strategy for soil quality improvement and resource recycling in sustainable agricultural systems. With the overuse of chemical fertilizers and ongoing degradation of arable land, reducing fertilizer inputs while maintaining crop yields and improving soil health has become a core challenge for modern agriculture (Wang et al., 2025b). As an abundant renewable organic resource, crop straw is rich in carbon (C), nitrogen (N), phosphorus (P), and potassium (K). Appropriate straw incorporation promotes nutrient cycling, enhances cropland carbon sequestration, improves soil physicochemical properties, stimulates microbial activity, and increases crop productivity (Li et al., 2024a; Zhang et al., 2023). A recent five-year consecutive field study in a soybean system demonstrated that deep straw incorporation combined with moderate mineral fertilizer reduction can simultaneously maintain high legume crop yield, enhance soil organic carbon sequestration, and improve soil nitrogen and phosphorus availability, with a maximum yield increase of 18.36% and soil organic carbon (SOC) elevation of 25.72% relative to conventional fertilization management (Zhang et al., 2025). This long-term field evidence directly confirms that straw incorporation can serve as a feasible core strategy to reduce chemical fertilizer inputs without compromising productivity in legume grain and oilseed production systems, providing a critical reference for optimizing agronomic management in similar legume crops. The ecological effects of straw incorporation are strongly dependent on the return method. Common practices include conventional shredding and incorporation, surface mulching, and deep incorporation paired with decomposition accelerators (Liu et al., 2024). Among these methods, deep incorporation with a decomposition accelerator integrates mechanical deep tillage with microbial and enzymatic additives. This combination accelerates straw decomposition and organic matter mineralization, thereby promoting the formation and stability of soil aggregates (Zhao et al., 2024). Previous studies have shown that deep incorporation of straw with decomposition accelerators increases soil organic carbon (SOC) content, enhances the proportion and water stability of macroaggregates, and improves soil aeration and water-holding capacity (Zhang et al., 2024; Song et al., 2024c). Meanwhile, organic acids, polysaccharides, and humic substances released during straw decomposition activate extracellular enzymes and accelerate C, N, and P cycling, ultimately boosting soil fertility (Li et al., 2025a). Notably, a core bottleneck limiting the efficiency of straw incorporation is the high carbon-to-nitrogen (C/N) ratio of cereal straw, which commonly induces nitrogen limitation for soil microorganisms, slows straw decomposition, and hinders the stable accumulation of soil organic carbon (SOC). A latest study addressing this constraint confirmed that combining straw amendment with legume crops can effectively optimize soil C/N stoichiometry via symbiotic biological nitrogen fixation, simultaneously promoting straw degradation and enhancing both SOC and total nitrogen (TN) contents in bulk soil and across all aggregate size fractions, with significantly better performance than straw application alone or straw paired with grass cover crops (Wang et al., 2025a). Therefore, clarifying how different straw incorporation methods regulate soil structure, enzyme activities, and microbial communities is critical for the efficient utilization of straw resources and the reduction of chemical fertilizer inputs.

Soil aggregates are the fundamental units that determine soil structural stability and nutrient retention capacity, and their formation and stabilization are jointly driven by organic matter inputs and microbial activity (de Goede et al., 2025). Straw incorporation provides carbon substrates for aggregate formation, while microbial metabolites such as extracellular polysaccharides and proteins act as biological binding agents (Flemming et al., 2025). Deep straw incorporation further stabilizes soil structure by optimizing decomposition kinetics and accelerating microbially mediated aggregate formation (Islam et al., 2023). Moreover, the hierarchical distribution of organic carbon across aggregate size fractions is closely linked to microbial diversity, as different size classes provide distinct ecological niches for specialized functional microbial groups (Huang et al., 2024). Extracellular enzymes are the core catalysts of microbially mediated soil C, N, and P cycling, and their activities serve as sensitive indicators of soil nutrient turnover rates and microbial metabolic potential (Song et al., 2024a). Straw incorporation generally increases the activities of hydrolytic enzymes (e.g., β-1,4-glucosidase, cellulase, acid phosphatase) and oxidative enzymes (phenol oxidase, peroxidase), thereby promoting organic matter degradation and nutrient release (Li et al., 2025c). Different straw incorporation methods may drive divergent responses of soil enzyme activities by altering substrate supply, rhizosphere microenvironments, and microbial community composition (Yang et al., 2024; Guan et al., 2023). However, the coupled effects of deep straw incorporation and fertilizer reduction on soil enzyme activities, as well as their links to aggregate formation and nutrient retention, remain insufficiently validated under field conditions.

Soil microbial communities are the core drivers of soil ecosystem functioning, as their structure and functional potential regulate organic matter decomposition, nutrient cycling, and aggregate stabilization (Liu et al., 2023a). Straw incorporation supplies abundant carbon and energy sources for soil microbes, accelerating the succession of bacterial and fungal communities. Previous work has revealed that actinomycetes and Bacillus species play pivotal roles in straw decomposition and the formation of stable soil organic carbon (Gong et al., 2022). Meanwhile, fungal mycelial networks promote soil particle binding and contribute substantially to the formation of macroaggregates (Ji et al., 2024). Accordingly, different straw incorporation methods may influence crop yield and quality by reshaping the diversity and composition of bacterial and fungal communities, which in turn regulate extracellular enzyme activities and aggregate formation.

Peanut (*Arachis hypogaea* L.) is a major oilseed crop in China, whose yield and quality are highly dependent on rhizosphere soil ecological processes. In the Huang-Huai-Hai Plain, a core peanut-producing region of China, long-term intensive cultivation coupled with excessive fertilizer application has led to soil degradation, disrupted microbial communities, and increased environmental risks, posing a critical challenge for the sustainability of peanut production (Kang et al., 2023). There is thus an urgent need for optimized cultivation practices that integrate efficient straw utilization with reduced fertilizer inputs to balance crop productivity with agroecosystem health. In this study, we compared different wheat straw incorporation methods under both conventional and reduced fertilizer regimes in a three-year field trial. We systematically evaluated their effects on peanut yield, soil aggregate composition and water stability, extracellular enzyme activities, and rhizosphere microbial diversity and function. Our primary goal was to elucidate the regulatory pathways linking straw incorporation methods to the cascade of “microbial community–enzyme activity–aggregate structure–nutrient retention–peanut yield”. We tested three hypotheses: (1) different straw incorporation methods significantly alter the structure and functional potential of rhizosphere bacterial and fungal communities; (2) deep straw incorporation with a decomposition accelerator enhances soil nutrient retention by increasing extracellular enzyme activities and promoting aggregate formation; (3) the combination of deep straw incorporation with reduced fertilizer input can maintain peanut yield, while supporting a more efficient, eco-friendly, and sustainable peanut production system. This research provides theoretical support for optimizing straw utilization and efficient peanut cultivation, as well as technical guidance for developing low-carbon, green, and sustainable cropping systems in the major agricultural regions of China.

## 2 Materials and methods

### 2.1 Experimental site

The three-year field experiment was conducted from 2022 to 2024 in Zhengyang County, Henan Province, China (32°27′37″ N, 114°20′30″ E). The study area has a warm-temperate monsoon climate with four distinct seasons, a mean annual temperature of approximately 15.0 °C, and a mean annual precipitation of ~920 mm, the majority of which falls between June and September. The tested soil is a lime concretion vertisol with a clay texture. Prior to the experiment, the 0–20 cm topsoil layer contained 17.5 g·kg^-^¹ organic matter, 1.08 g·kg^-^¹ total N, 0.68 g·kg^-^¹ total P, 18.1 g·kg^-^¹ total K, and had a pH of 4.9. The cropping system at the site is a long-term winter wheat–peanut rotation, with uniform soil fertility, reliable irrigation and drainage conditions, and no history of contamination, ensuring the representativeness and reproducibility of the experiment. The preceding winter wheat crop was the local commercial cultivar “Bainong 207”, and the peanut cultivar used for all experimental years was “Yuanza 9102”.

### 2.2 Experimental design and management

The experiment was arranged in a randomized complete block design (RCBD)​ with six treatments and three replicate blocks. Each block was considered a replication, and within each block, all six treatments were randomly assigned to individual plots (12 m × 5 m). The six treatments were as follows:

CT: No straw return + conventional fertilization

SI: Conventional straw incorporation + conventional fertilization

SD: Deep straw incorporation with decomposition accelerator + conventional fertilization

SDR: Deep straw incorporation with decomposition accelerator + 25% fertilizer reduction

SM: Surface straw mulching + conventional fertilization

SMR: Surface straw mulching + 25% fertilizer reduction

The chemical fertilizers used were urea (46% N), single superphosphate (12% P_2_O_5_), and potassium sulfate (45% K_2_O). Wheat straw was applied at a rate of 7.5 t·ha^-^¹ (dry matter basis), chopped to a particle size of 2–5 cm with a moisture content of 12%. The elemental composition of the straw was 45.2% C, 0.68% N, 0.08% P, and 1.12% K (mean of three subsamples). The decomposition accelerator used (“Wangnongbao”, Zhengzhou Wonobio Biotechnology Co., Ltd.) contained Bacillus subtilis (1×10^9^ CFU·g^-^¹), Trichoderma harzianum (5×10^8^ CFU·g^-^¹), cellulase (100 U·g^-^¹), and lignin peroxidase (50 U·g^-^¹), applied at a rate of 3 kg·ha^-^¹. The accelerator was premixed with 20 kg·ha^-^¹ wheat bran, broadcast evenly over the soil surface, and incorporated into the 20–30 cm soil layer with chopped straw via rotary tillage to accelerate straw decomposition. The total straw application rate was determined based on the grain yield of the preceding wheat crop. After chopping, all straw was fully incorporated into the soil or applied as surface mulch according to the treatment specifications. For deep incorporation treatments, straw was premixed with the decomposition accelerator and incorporated into the 30 cm soil layer via deep tillage to enhance decomposition. For mulching treatments, chopped straw was evenly applied to the surface of the plow layer.

All fertilizers were applied as a basal dose prior to peanut sowing. The conventional fertilization rate was 150 kg N ha^-^¹, 120 kg P_2_O_5_ ha^-^¹, and 150 kg K_2_O ha^-^¹; reduced-fertilizer treatments received 75% of these conventional rates for each nutrient. All fertilizers were applied pre-sowing, followed by rotary tillage for seedbed preparation.

Peanuts were sown in raised ridges, with each ridge approximately 75 cm wide and 18 cm high. Two rows of peanuts were sown per ridge, with a row spacing of 25 cm and a plant spacing of 15 cm. Two seeds were sown per hole at a depth of 4–5 cm, resulting in a planting density of approximately 3.56×10^5^ plants ha^-^¹. All field management practices, including weeding, irrigation, and pest and disease control, followed local high-yield peanut production standards to ensure uniformity across treatments.

### 2.3 Soil collected and analysis

Soil samples were collected from the 0–30 cm soil layer at the peanut pod-filling stage. For each plot, five random soil cores were collected in an S-shaped pattern and composited into a single representative sample (~2 kg fresh weight). Each composite sample was split into two subsamples: one was air-dried for analysis of soil physicochemical properties and aggregate composition, and the other was divided and stored at 4 °C and −80 °C for extracellular enzyme activity assays and microbial community analysis, respectively.

Air-dried soil samples were ground to pass through a 0.15 mm sieve, and soil organic matter, total nutrients, and available nutrients were analyzed using standard agrochemical analysis methods. Dissolved organic carbon (DOC) and microbial biomass carbon (MBC) were determined using the chloroform fumigation–extraction method with 0.5 M K_2_SO^4^. Total dissolved carbon (TDC) was quantified using a total organic carbon (TOC) analyzer (Shimadzu TOC-L, Japan) (Liu et al., 2023b). Total N and total P were determined colorimetrically after acid digestion, and available phosphorus (AP) was extracted with 0.5 M NaHCO_3_ (pH 8.5) and quantified via molybdenum-antimony colorimetry. Rhizosphere soil samples were collected at the peanut pod-filling stage. The activities of eight extracellular enzymes were assayed: β-1,4-glucosidase (BG), β-xylosidase (BX), cellobiohydrolase (CBH), β-N-acetylglucosaminidase (NAG), leucine aminopeptidase (LAP), acid phosphatase (ACP), peroxidase (POD), and polyphenol oxidase (PPO). Soil extracellular enzyme activities were determined using a microplate fluorimetric assay with 4-methylumbelliferone (MUB)- and 7-amino-4-methylcoumarin (AMC)-linked substrates, following established methods (German et al., 2012; Moorhead et al., 2016). Fluorescence was measured using a microplate reader (BioTek Synergy H1) with excitation/emission wavelengths set at 365/450 nm for MUB-based substrates and 360/450 nm for AMC-based substrates (a commonly used setting within the typical range of 355–380 nm excitation and 440–460 nm emission for AMC). Blank controls and quench controls were included in all assays. Enzyme activities were expressed per unit mass of dry soil.

Soil aggregates were separated into size fractions using the wet-sieving method described by Sekaran (Sekaran et al., 2021). Briefly, air-dried soil samples were pre-soaked in deionized water for 10 min, then vertically oscillated at 30 cycles min^-^¹ for 30 min through a series of sieves (2, 1, 0.5, and 0.25 mm) to separate five aggregate size classes: >2 mm, 2–1 mm, 1–0.5 mm, 0.5–0.25 mm, and<0.25 mm. The mean weight diameter (MWD) and geometric mean diameter (GMD) of soil aggregates were calculated using the following formulas:


$$\text{MWD}=\sum_{i=1}^{n}{\bar{x}}_{i}w_{i}$$

$$\text{GMD}=\exp\left(\frac{\sum_{i=1}^{n}w_{i}\ln{\bar{x}}_{i}}{\sum_{i=1}^{n}w_{i}}\right)$$

Where ${\bar{x}}_{i}$ is the mean diameter of the i-th aggregate class (mm), and *w_i_* is the mass proportion of the i-th class (%). The proportion of water-stable aggregates ≥0.25 mm (*R_0_._25_*) was also calculated as an indicator of aggregate stability.

### 2.4 Soil microbial community analysis

Total genomic DNA was extracted from rhizosphere soil samples using the DNeasy PowerSoil Kit (Qiagen, Hilden, Germany) following the manufacturer’s instructions. The V3–V4 hypervariable region of the bacterial 16S rRNA gene was amplified using the primer pair 338F/806R, and the fungal ITS1 region was amplified using the primer pair ITS1F/ITS2. The V3–V4 hypervariable region of the bacterial 16S rRNA gene was amplified using the universal primer pair 338F (5′-ACTCCTACGGGAGGCAGCAG-3′) and 806R (5′-GGACTACHVGGGTWTCTAAT-3′) (Caporaso et al., 2011). The fungal ITS1 region was amplified using the primer pair ITS1F (5′-CTTGGTCATTTAGAGGAAGTAA-3′) and ITS2 (5′-GCTGCGTTCTTCATCGATGC-3′) (Gardes and Bruns, 1993). High-throughput sequencing was performed on the Illumina NovaSeq 6000 platform (2 × 250 bp paired-end reads).

Raw sequences were quality-filtered, denoised, merged, and chimera-checked using the DADA2 plugin in QIIME2. Amplicon Sequence Variants (ASVs) with a total abundance of less than 10 reads across all samples were filtered out to remove potential spurious sequences. Taxonomic assignment was performed using the SILVA (v138) database for bacteria and the UNITE (v9.0) database for fungi. Fungal functional guilds were annotated using the FUNGuild database. Bacterial functional profiles were predicted using PICRUSt2 based on 16S rRNA gene sequences, and Cluster of Orthologous Groups (COG) functional classification was performed to identify key metabolic pathways. Alpha diversity indices (Chao1 richness estimator) and beta diversity metrics (principal coordinates analysis [PCoA], non-metric multidimensional scaling [NMDS] based on Bray–Curtis dissimilarity) were calculated to assess microbial community diversity and structure. Co-occurrence networks were constructed using SparCC correlations, with a threshold of |r| > 0.6 and *P* < 0.01 for significant correlations.

### 2.5 Plant sampling and yield measurement

Ten representative peanut plants were collected from each plot at the pegging stage and maturity stage, respectively. Plant samples were separated into roots, shoots, and pods, oven-dried at 75 °C to constant weight, ground, and sieved through a 0.15 mm sieve. The concentrations of N, P, and K in plant tissues were determined via Kjeldahl digestion, molybdenum–antimony colorimetry, and flame photometry, respectively.

Peanut yield and yield components, including 100-pod weight, shelling percentage, and total pod yield, were measured annually from 2022 to 2024. At physiological maturity, all peanut plants in the designated harvest area of each plot were hand-harvested, air-dried, and weighed. Final pod yield was expressed as kg ha^-^¹.

### 2.6 Statistical analysis

All statistical analyses were performed in R (v4.3.2). Linear mixed-effects models were used to analyze the data, with treatment and year included as fixed effects, and block nested within year as a random effect. The treatment × year interaction was tested in the full model; when the interaction was non-significant, the model was refit without the interaction term, and marginal means with Tukey’s HSD adjusted pairwise contrasts were reported. The normality and homoscedasticity of model residuals were checked prior to analysis.

For yield traits, soil physicochemical properties, enzyme activities, and nutrient indices, the normality of data distribution was tested using the Shapiro–Wilk test, and homoscedasticity was verified using Levene’s test. One-way analysis of variance (ANOVA) was used to test for significant treatment effects, and means were separated using Tukey’s HSD *post-hoc* test at a significance level of α = 0.05. Non-normally distributed data were analyzed using the non-parametric Kruskal–Wallis test. Results are presented as mean ± standard error (SE; n = 3 biological replicates). Significant differences between treatments are indicated by different lowercase letters (*P* < 0.05).

## 3 Results

### 3.1 Effects of wheat straw incorporation regimes on peanut yield traits

Straw incorporation methods significantly altered peanut 100-pod weight, shelling percentage, and total pod yield across the three-year experiment (**Table 1**). Across all years, deep straw incorporation with a decomposition accelerator (SD and SDR treatments) exhibited the best performance for all measured yield traits. Specifically, the SD treatment consistently produced the highest 100-pod weight and total yield. In 2022, SD increased 100-pod weight by 8.3% and total yield by 16.3% relative to the no-straw control (CT). Although yield metrics for SDR were slightly lower than those for SD, the difference was not statistically significant (*P* > 0.05), indicating that deep straw incorporation with a decomposition accelerator can maintain high peanut yields despite a 25% reduction in fertilizer input.

**Table 1:** Effects of wheat straw incorporation regimes on peanut yield traits (2022–2024).

Year	Treatment	100-pod weight (g)	Shelling percentage (%)	Yield (kg ha^-^¹)
2022	CT	206.43 ± 6.77 bc	73.20 ± 0.82 a	5301.75 ± 504.15 b
	SI	215.65 ± 3.62 ab	71.26 ± 1.37 b	4412.40 ± 418.80 cd
	SD	223.57 ± 16.18 a	73.09 ± 0.76 a	6173.40 ± 614.85 a
	SDR	219.94 ± 11.99 a	73.68 ± 0.68 a	5784.15 ± 477.45 ab
	SM	199.64 ± 12.65 bc	70.70 ± 1.03 b	4988.25 ± 474.00 bc
	SMR	193.60 ± 3.29 c	73.47 ± 1.10 a	4103.40 ± 169.05 d
2023	CT	183.20 ± 12.46 c	70.22 ± 1.50 b	4270.95 ± 215.70 c
	SI	190.71 ± 6.13 bc	70.08 ± 1.84 b	4542.15 ± 279.30 bc
	SD	213.98 ± 6.40 a	73.35 ± 0.70 a	5171.25 ± 221.10 a
	SDR	207.11 ± 13.14 ab	71.47 ± 0.62 ab	4813.50 ± 326.25 ab
	SM	191.62 ± 20.18 bc	72.10 ± 1.33 ab	4695.60 ± 201.90 b
	SMR	183.48 ± 8.76 c	67.43 ± 1.93 c	4197.60 ± 108.45 c
2024	CT	203.03 ± 1.10 b	74.75 ± 1.03 bc	5075.85 ± 308.70 b
	SI	207.15 ± 8.28 ab	76.27 ± 1.20 ab	5309.25 ± 540.40 ab
	SD	213.67 ± 2.25 a	76.62 ± 0.37 a	5729.55 ± 126.75 a
	SDR	211.31 ± 3.21 ab	75.07 ± 1.28 abc	5567.25 ± 307.50 ab
	SM	210.84 ± 3.01 ab	76.36 ± 0.45 ab	5169.30 ± 320.10 ab
	SMR	202.39 ± 6.81 b	74.29 ± 0.90 c	4708.95 ± 436.95 c

Values are means ± SE (n=3 plots per treatment in each year). Within each year, means followed by different lowercase letters in the same column differ significantly at *P* < 0.05 (one-way ANOVA, Tukey’s HSD test).

This trend was consistent across the 2023 and 2024 growing seasons: SD and SDR resulted in significantly higher 100-pod weight and pod yield than the conventional incorporation (SI) and surface mulching (SM) treatments, and far greater values than the CT and mulching with fertilizer reduction (SMR) treatments. Shelling percentage varied relatively little among treatments, with a three-year mean ranging from 70% to 74%, suggesting that straw incorporation method had limited effects on kernel plumpness; nevertheless, SD and SDR showed a slight trend of higher shelling percentage relative to other treatments. Across the three-year study period, SD and SDR maintained the most sTable 100-pod weight and yield performance, with average yields 20–30% higher than CT. Notably, despite the 25% fertilizer reduction, SDR achieved yields statistically equivalent to SD (*P* > 0.05), highlighting the high nutrient use efficiency and yield stability of the deep incorporation system under reduced fertilizer input.

Although absolute yield values varied between years due to climatic conditions, the relative performance ranking of the treatments and the significant yield advantages of SD and SDR over CT, SI, and SM/SMR were consistent across all three experimental years.​ This demonstrates the robust and reproducible effect of deep straw incorporation with a decomposition accelerator on enhancing peanut yield.

### 3.2 Effects of wheat straw incorporation regimes on soil aggregate composition and water stability

Straw incorporation methods significantly altered soil aggregate size distribution and water stability (**Table 2**). Deep straw incorporation with a decomposition accelerator (SD and SDR) outperformed all other treatments, producing the highest *R_0_._25_*, MWD, and GMD values. Specifically, the SD treatment had the highest *R_0_._25_* value (0.684), representing a 26.4% increase relative to CT. The *R_0_._25_* value for SDR (0.672) was statistically equivalent to that of SD (non-significant, *P* > 0.05). The SI and SM treatments slightly increased *R_0_._25_* relative to CT, but their values remained significantly lower than those of SD and SDR.

**Table 2:** Effects of wheat straw incorporation regimes on soil aggregate water stability indicators.

Treatment	*R*_0.25_	MWD (mm)	GMD (mm)
CT	0.541 ± 0.096 b	0.778 ± 0.201 b	0.387 ± 0.079 b
SI	0.604 ± 0.090 a	1.108 ± 0.162 a	0.481 ± 0.085 a
SD	0.684 ± 0.011a	1.542 ± 0.241 a	0.629 ± 0.064 a
SDR	0.672 ± 0.036 a	1.397 ± 0.206 a	0.595 ± 0.156 a
SM	0.593 ± 0.020 a	0.825 ± 0.111 b	0.420 ± 0.030 b
SMR	0.609 ± 0.032 a	0.921 ± 0.075 b	0.490 ± 0.036 a

Values are mean ± SE (n = 3). Different lowercase letters within the same column indicate significant differences at *P* < 0.05 (one-way ANOVA, Tukey’s HSD test).

For MWD, the SD and SDR treatments had significantly higher values than all other treatments (*P* < 0.05), with SD recording the highest MWD (1.542 mm), an increase of ~98.3% compared with CT. This result demonstrates that deep straw incorporation with accelerated decomposition substantially enhances soil aggregate stability, consistent with the trend observed for *R_0_._25_*, and suggests that deep incorporation promotes the production of organic binding agents and enhances aggregate cohesion. GMD values were also highest under SD and SDR (0.629 mm and 0.595 mm, respectively), which were significantly greater than those under CT and SM (*P* < 0.05). Larger GMD values indicate a shift toward larger, more stable aggregate size classes, reflecting improved structural uniformity and stability of the soil under deep straw incorporation.

### 3.3 Effects of wheat straw incorporation regimes on soil extracellular enzyme activities

**Figure 1:**
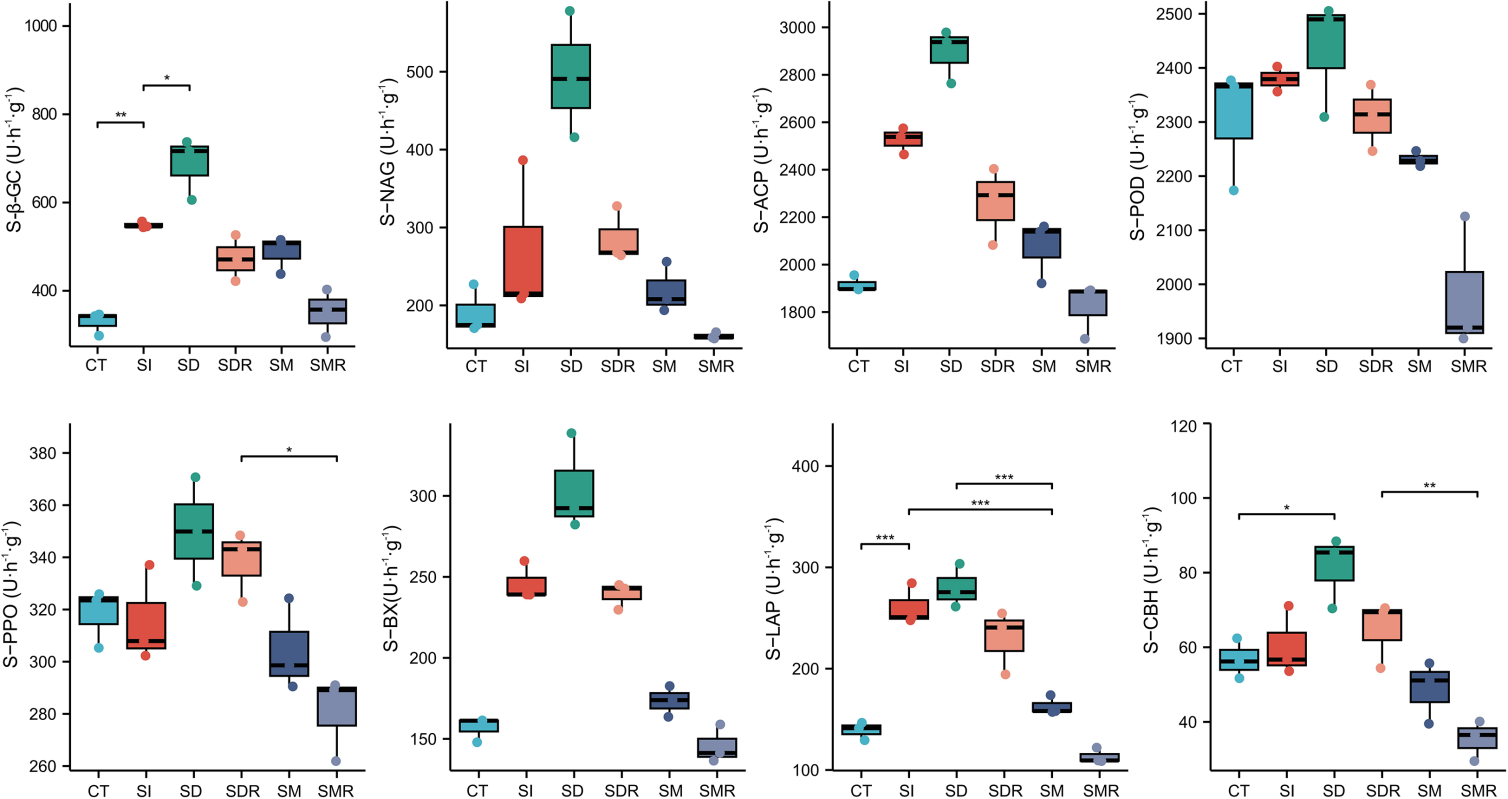
Effects of wheat straw incorporation regimes on soil extracellular enzyme activities. Boxplots show the activities of C-, N-, and P-cycling hydrolytic enzymes (β-1,4-glucosidase, BG; β-xylosidase, BX; cellobiohydrolase, CBH; β-N-acetylglucosaminidase, NAG; leucine aminopeptidase, LAP; acid phosphatase, ACP) and oxidative enzymes (peroxidase, POD; polyphenol oxidase, PPO) across the six experimental treatments. Boxes represent the median and interquartile range (IQR), whiskers extend to 1.5× the IQR, and individual points represent biological replicates (n = 3). Asterisks denote significant differences among treatments at *P* < 0.05 (one-way ANOVA with Tukey’s HSD *post-hoc* test). Enzyme activity units: U·h^-^¹·g^-^¹ dry soil. *: P < 0.05, **: P < 0.01, ***: P < 0.001.

Straw incorporation methods significantly affected the activities of all measured soil extracellular enzymes (*P* < 0.05), with the overall activity ranking across treatments as follows: SD > SDR > SI > SM > CT > SMR (**Figure 1**). Mean enzyme activity data for all treatments are presented in **Supplementary Table 1**. Relative to CT, SD and SDR significantly increased the activities of C-, N-, and P-cycling hydrolytic enzymes, as well as oxidative enzymes. Specifically, relative to CT, SD treatment increased the activities of BG by 2.1-fold, CBH by 1.4-fold, NAG by 2.6-fold, LAP by 2-fold, ACP by 1.5-fold. Although there was no significant difference between POD and PPO (*P* > 0.05), there was a trend toward elevation. SDR treatment maintained equivalent enzyme activities to SD, with no significant difference between the two treatments (*P* > 0.05).

The enhanced enzyme activities under deep incorporation treatments likely reflect stimulated microbial metabolism and increased secretion of polysaccharide- and protein-degrading enzymes, which accelerate organic matter decomposition and C/N/P cycling, thereby improving soil nutrient transformation and supply capacity. Meanwhile, elevated oxidative enzyme activities may facilitate lignin humification and the stabilization of soil organic carbon. Notably, despite the 25% fertilizer reduction, SDR maintained extracellular enzyme activities comparable to those of SD (non-significant, *P* > 0.05). This result suggests that deep straw incorporation with a decomposition accelerator not only promotes efficient straw decomposition, but also sustains robust microbial function and nutrient cycling, reducing the reliance on chemical fertilizer inputs. This practice thus represents a key tillage strategy for achieving eco-friendly yield improvement and sustainable agricultural development.

### 3.4 Shifts in rhizosphere carbon pools and nutrient distribution

**Figure 2:**
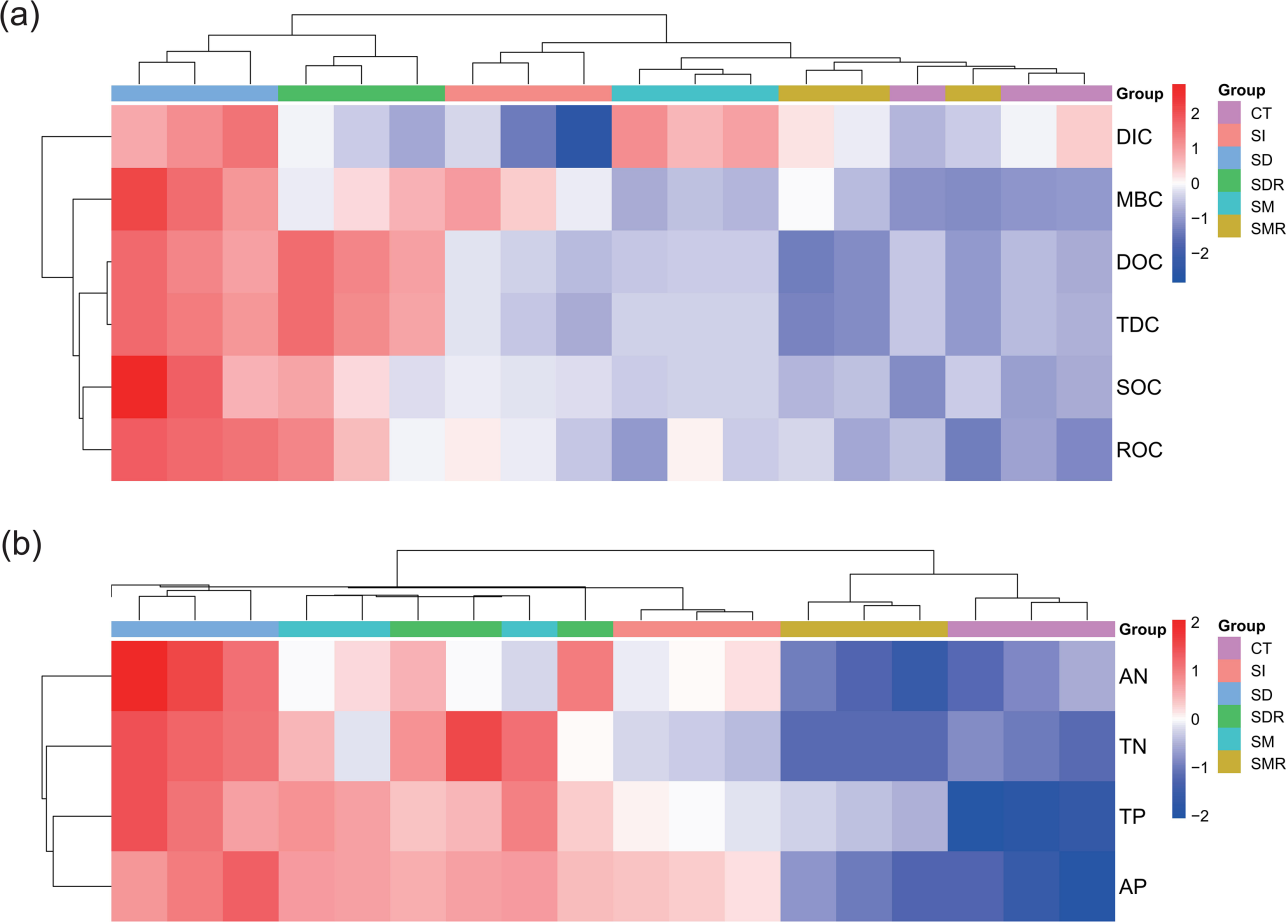
Hierarchical clustering heatmaps of rhizosphere carbon pool and soil nutrient indicators. **(A)** Heatmap of carbon pool indicators: dissolved inorganic carbon (DIC), dissolved organic carbon (DOC), total dissolved carbon (TDC), microbial biomass carbon (MBC), soil organic carbon (SOC), and recalcitrant organic carbon (ROC). **(B)** Heatmap of soil nutrient indicators: available nitrogen (AN), total nitrogen (TN), available phosphorus (AP), and total phosphorus (TP). Values are z-score standardized per variable to highlight relative differences among treatments; hierarchical clustering was performed for both samples and variables. The color gradient represents relative abundance from low (blue) to high (red).

Hierarchical clustering heatmaps revealed distinct grouping patterns of rhizosphere carbon pool and nutrient indicators across the different straw incorporation treatments (**Figure 2**). For the carbon pool indicators, DOC, TDC, and MBC clustered into one group, while soil organic carbon (SOC) and recalcitrant organic carbon (ROC) formed a separate cluster. The samples were clearly separated into two major branches: a high-value branch dominated by the SD and SDR treatments, and a low-value branch dominated by CT, SI, SM, and SMR (**Figure 2A**). Overall, SD and SDR exhibited significantly higher DOC, TDC, and MBC contents, with SOC and ROC also showing a trend of elevation under these two treatments. These results indicate that deep straw incorporation increases the content of rhizosphere labile active carbon fractions, while also promoting the accumulation of stable organic carbon pools. Dissolved inorganic carbon (DIC) showed minimal variation among treatments and did not follow the distribution pattern of the other carbon fractions.

For soil nutrient indicators, values were consistently higher under SD and SDR, particularly for available nitrogen (AN) and total nitrogen (TN). AP and total phosphorus (TP) were also concentrated in the high-value zones under SD and SDR, while CT, SI, SM, and SMR tended to fall into the low-value zones (**Figure 2B**). In both carbon pool and nutrient heatmaps, SD and SDR clustered within the same high-value branch, while CT, SI, and the majority of SM and SMR samples clustered within the low-value branch. This pattern demonstrates that deep straw incorporation with a decomposition accelerator—especially the SDR treatment, which combines deep incorporation, decomposition accelerator, and 25% fertilizer reduction—consistently enhances indicators along both the “labile C–microbial biomass–stable C” and “available N/P–total nutrient pools” pathways. In contrast, the no-straw control, conventional incorporation, and surface mulching treatments generally resulted in lower levels of these carbon and nutrient indicators.

Collectively, these findings suggest that deep straw incorporation accelerates straw decomposition, reshapes the rhizosphere carbon pool structure toward higher lability and stability, and enhances soil N and P availability and total nutrient pool capacity. These improvements provide the material foundation for the subsequent increases in peanut yield observed in this study.

### 3.5 Responses of rhizosphere bacterial communities to straw incorporation regimes

**Figure 3:**
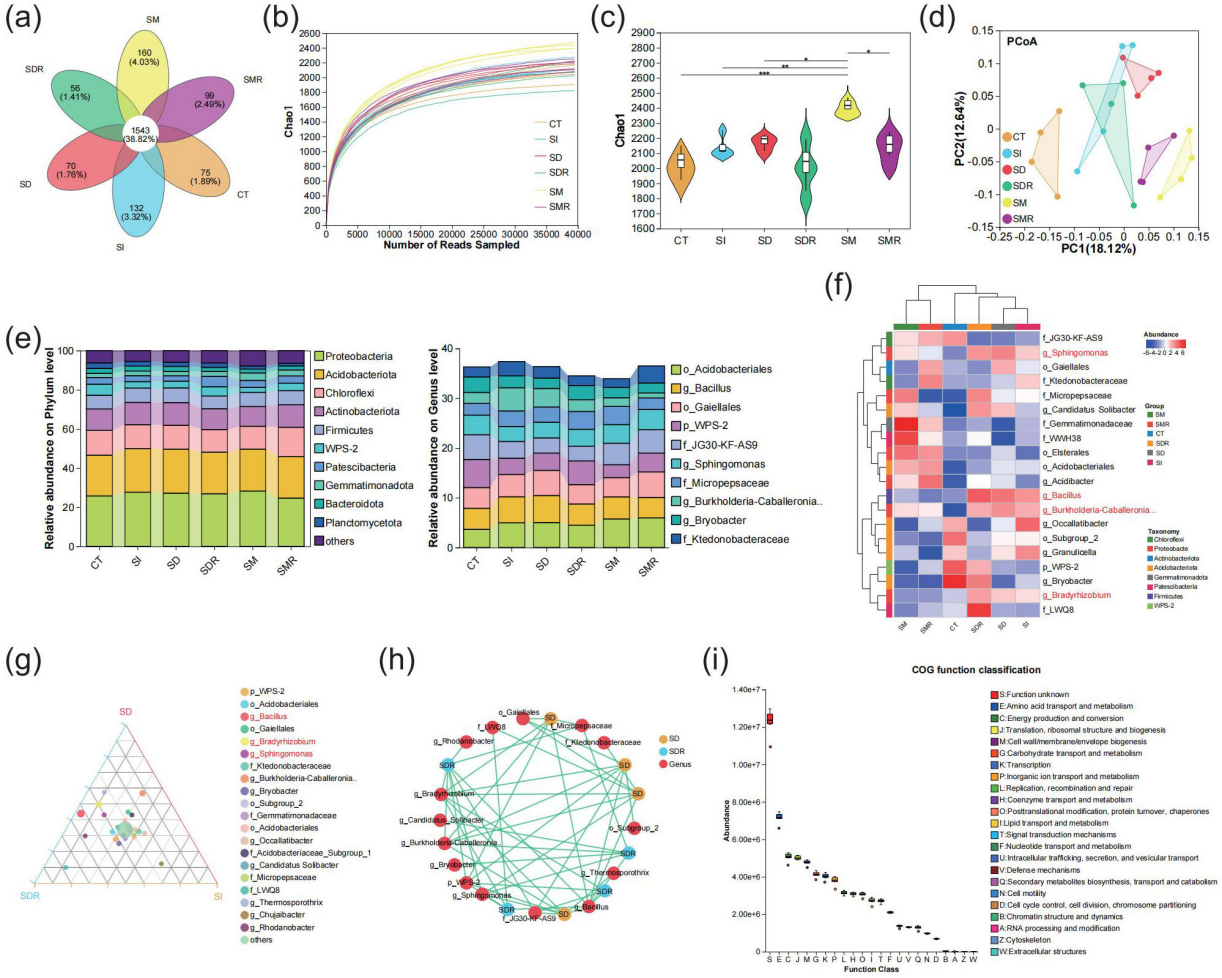
Responses of rhizosphere bacterial communities to wheat straw incorporation regimes. **(A)** Venn diagram showing shared and treatment-specific amplicon sequence variants (ASVs) across treatments. **(B)** Rarefaction curves of the Chao1 richness estimator, demonstrating sufficient sequencing depth. **(C)** Alpha diversity (Chao1 index) across treatments; bars show mean ± SE (n = 4 biological replicates per treatment), and asterisks indicate significant differences at *P* < 0.05 (one-way ANOVA with Tukey’s HSD). **(D)** Principal coordinates analysis (PCoA) based on Bray–Curtis dissimilarity, showing the separation of bacterial community structures among treatments. **(E)** Relative abundance of bacterial communities at the phylum level (left) and genus level (right) (stacked bars represent the mean of n = 4 replicates). **(F)** Hierarchical clustering heatmap of dominant bacterial genera (values are row-scaled). **(G)** Ternary plot showing the partitioning of dominant bacterial genera across the SI, SD, and SDR treatments. **(H)** Co-occurrence network of dominant bacterial genera for the SD and SDR treatments; nodes represent genera, and edge thickness reflects the strength of SparCC correlations (|r| > 0.6, *P* < 0.01, Benjamini–Hochberg corrected). **(I)** COG functional category profiling of bacterial communities in the SD and SDR treatments. *: P < 0.05, **: P < 0.01, ***: P < 0.001.

Rhizosphere bacterial communities showed significant structural and functional differentiation among the different straw incorporation treatments (**Figure 3**). Although 1,543 core ASVs (accounting for 38.8% of total ASVs) were shared across all treatments, a large number of treatment-specific unique ASVs were also detected. This finding indicates that straw incorporation not only alters the overall composition of the bacterial community, but also drives treatment-specific shifts in bacterial taxa (**Figure 3A**). Rarefaction curves for the Chao1 richness estimator reached a clear plateau, confirming that the sequencing depth was sufficient to capture the majority of bacterial diversity in the samples, and that the sequencing results were reliable for downstream analysis (**Figure 3B**).

Analysis of alpha diversity revealed that the SD, SDR, and SM treatments had significantly higher Chao1 indices (a measure of community richness) than CT, SI, and SMR (P< 0.05), with SM exhibiting the highest overall richness (**Figure 3C**). This result demonstrates that different straw incorporation methods can significantly increase rhizosphere bacterial richness, with surface mulching and deep incorporation with a decomposition accelerator having the most pronounced effects. Principal coordinates analysis (PCoA) based on Bray–Curtis dissimilarity further confirmed the distinct separation of bacterial community structures among treatments. All straw incorporation treatments (SI to SMR) were clearly separated from the no-straw control (CT), and distinct clustering was also observed among the different straw return methods (**Figure 3D**). These results indicate that straw incorporation method is a key driver shaping the structure of rhizosphere bacterial communities.

At the phylum level, the dominant bacterial taxa across all samples included Proteobacteria, Actinobacteria, Acidobacteria, and Chloroflexi. At the genus level, the dominant genera included *Bacillus*, *Sphingomonas*, *Burkholderia-Caballeronia-Paraburkholderia*, and *Bryobacter* (**Figure 3E**). Hierarchical clustering heatmaps and ternary plots consistently revealed that *Bacillus*, *Sphingomonas*, and *Burkholderia-Caballeronia-Paraburkholderia* were significantly enriched in the SD and SDR treatments (**Figures 3F, G**). This pattern suggests that deep straw incorporation combined with a decomposition accelerator selectively enriches bacterial taxa involved in organic matter degradation and plant growth promotion.

Co-occurrence network analysis for the SD and SDR treatments revealed that key genera including *Bacillus*, *Sphingomonas*, and *Bradyrhizobium* had the highest node degrees and betweenness centrality values, indicating their core positions in the microbial interaction network (**Figure 3H**). These findings suggest that deep straw incorporation may enhance the complexity of microbial co-occurrence networks and the robustness of the bacterial community via these keystone taxa. COG functional profiling further showed that bacterial communities under SD and SDR were primarily enriched in metabolic pathways related to carbohydrate metabolism, amino acid metabolism, and energy metabolism (**Figure 3I**). This result indicates that deep straw incorporation with accelerated decomposition enhances the metabolic activity and nutrient transformation potential of rhizosphere bacterial communities.

### 3.6 Responses of rhizosphere fungal communities to straw incorporation regimes

**Figure 4:**
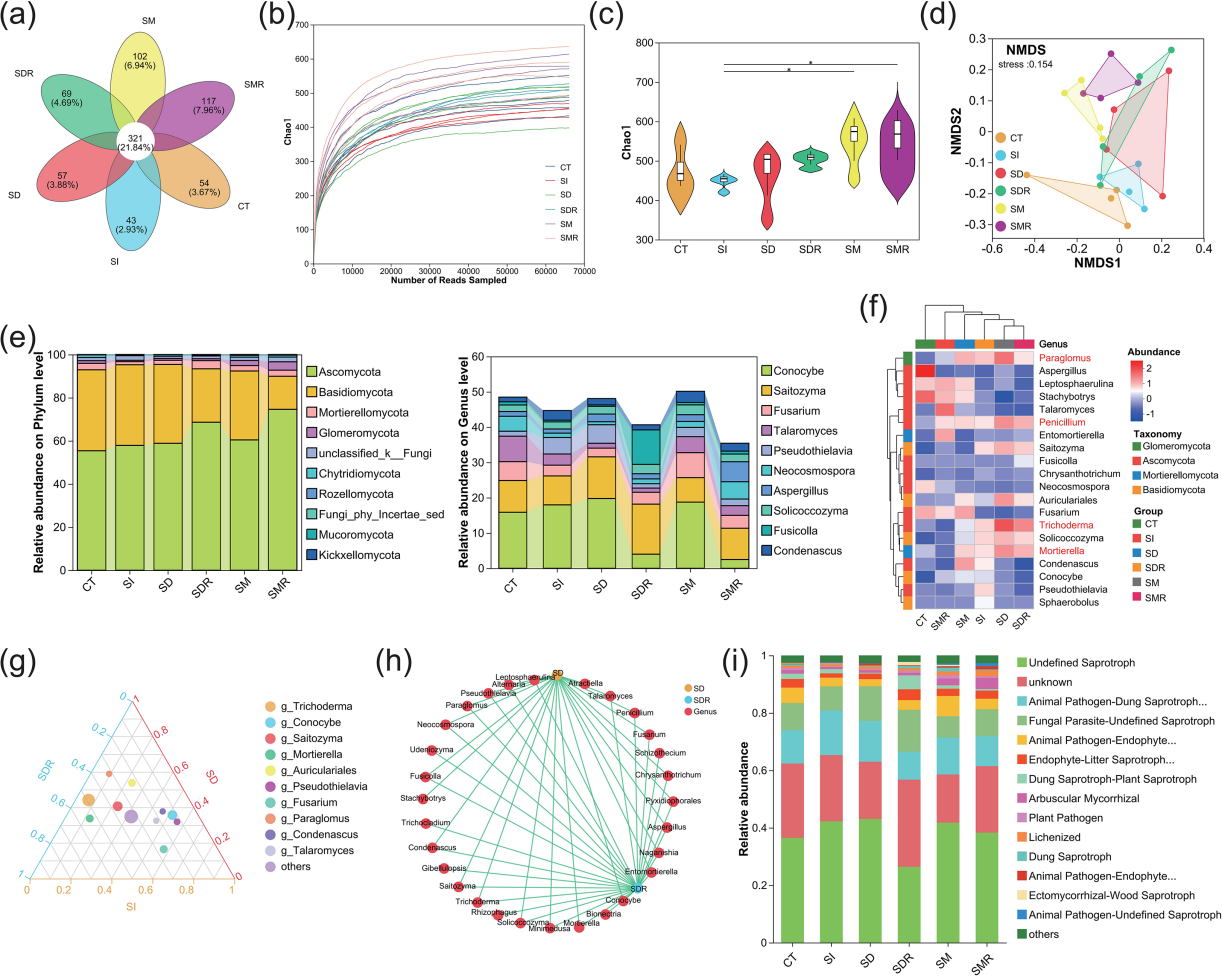
Responses of rhizosphere fungal communities to wheat straw incorporation regimes. **(A)** Venn diagram showing shared and treatment-specific ASVs across treatments. **(B)** Rarefaction curves of the Chao1 richness estimator, indicating sufficient sequencing depth. **(C)** Alpha diversity (Chao1 index) across treatments; bars show mean ± SE (n = 4 biological replicates per treatment), and asterisks denote significant differences at *P* < 0.05 (one-way ANOVA with Tukey’s HSD). **(D)** Non-metric multidimensional scaling (NMDS) ordination based on Bray–Curtis dissimilarity (stress = 0.154), showing the separation of fungal community structures among treatments. **(E)** Relative abundance of fungal communities at the phylum level (left) and genus level (right) (stacked bars represent the mean of n = 4 replicates). **(F)** Hierarchical clustering heatmap of dominant fungal genera (values are row-scaled). **(G)** Ternary plot showing the partitioning of dominant fungal genera across the SI, SD, and SDR treatments. **(H)** Co-occurrence network of dominant fungal genera for the SD and SDR treatments; nodes represent genera, and edge thickness reflects the strength of SparCC correlations (|r| > 0.6, *P* < 0.01, Benjamini–Hochberg corrected). **(I)** Relative abundance of FUNGuild functional guilds for fungal communities in the SD and SDR treatments. *P < 0.05.

Rhizosphere fungal communities exhibited significant differences in composition, diversity, and functional potential across the different straw incorporation treatments (**Figure 4**). Although 321 core ASVs (21.8% of total ASVs) were shared among all treatments, a large number of treatment-specific unique ASVs were identified. Treatments with surface straw mulching (SM and SMR) harbored the highest number of unique fungal ASVs, consistent with the pattern observed for bacterial diversity (**Figure 4A**). Rarefaction curves reached a clear plateau, indicating that the sequencing depth was adequate to capture the majority of fungal diversity in the samples (**Figure 4B**).

Fungal alpha diversity (Chao1 index) was significantly higher under SD and SDR than under CT and SI, with the highest Chao1 values observed in the SM and SMR treatments (*P* < 0.05) (**Figure 4C**). This trend is consistent with the bacterial alpha diversity results, confirming that straw addition generally enhances rhizosphere fungal richness. Non-metric multidimensional scaling (NMDS) based on Bray–Curtis dissimilarity revealed clear separation of fungal community structures among treatments, with the SD/SDR cluster and SM/SMR cluster distinctly separated from the no-straw control (CT). The stress value of the NMDS ordination was 0.154 (< 0.2), indicating a good and reliable fit of the ordination (**Figure 4D**). These results confirm that different straw incorporation methods significantly shape the structure of rhizosphere fungal communities.

At the phylum level, the dominant fungal taxa across all samples included Ascomycota, Basidiomycota, and Mortierellomycota. At the genus level, saprotrophic and beneficial fungal genera such as *Trichoderma*, *Mortierella*, and *Saitozyma* were significantly enriched under SD and SDR. In contrast, potential phytopathogenic genera (e.g., *Fusarium*, *Aspergillus*) had higher relative abundances under the no-straw control and surface mulching treatments (**Figure 4E**). Hierarchical clustering heatmaps and ternary plots consistently showed that key beneficial genera including *Trichoderma*, *Mortierella*, *Paraglomus*, and *Penicillium* were more abundant in the deep incorporation treatments (SD and SDR). Conversely, pathogenic genera such as *Fusarium* and *Aspergillus* were enriched under the no-straw control and mulching treatments (**Figures 4F, G**).

Genus-level co-occurrence network analysis for SD and SDR identified *Trichoderma* and *Mortierella* as core keystone nodes with the highest connectivity, suggesting that deep straw incorporation enhances microbial synergistic interactions and the ecological stability of the fungal community (**Figure 4H**). FUNGuild functional annotation further revealed that deep straw incorporation significantly increased the relative abundance of saprotrophic fungi, while reducing the relative abundance of phytopathogenic fungal groups (*P* < 0.05) (**Figure 4I**). These findings collectively demonstrate that deep straw incorporation with a decomposition accelerator fosters a healthier, more functionally efficient rhizosphere fungal community.

### 3.7 Coupling relationships between rhizosphere microbial communities and soil nutrient cycling

**Figure 5:**
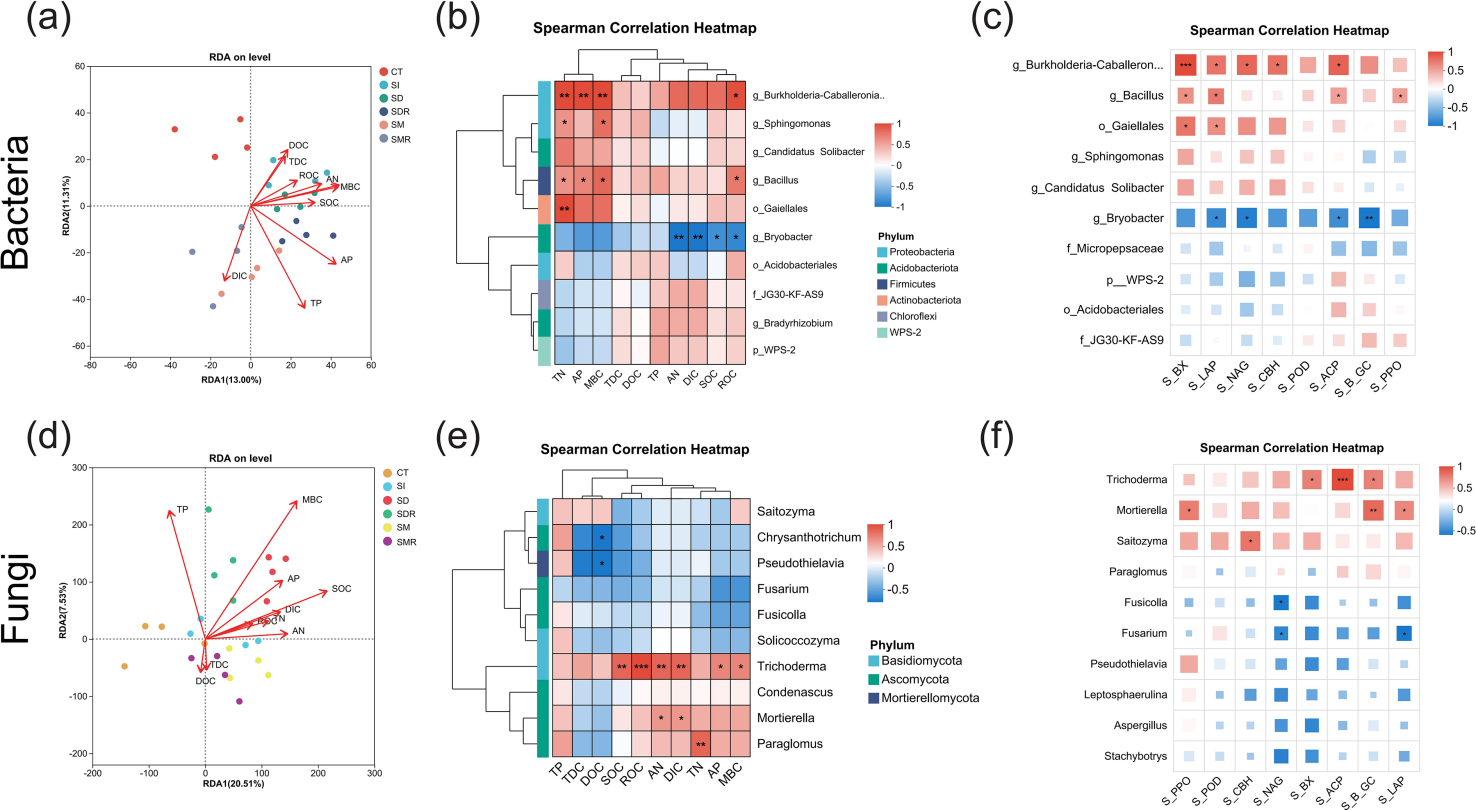
Coupling relationships between rhizosphere microbial communities, extracellular enzyme activities, and soil C/N/P pools across straw incorporation treatments. **(A)** Redundancy analysis (RDA) of bacterial communities constrained by soil carbon and nutrient indicators (DOC, TDC, MBC, SOC, AN, AP). **(B)** Spearman correlation heatmap between dominant bacterial genera and soil carbon/nutrient indicators (values are correlation coefficients; asterisks mark significant correlations after Benjamini–Hochberg correction: *P* < 0.05). **(C)** Spearman correlation heatmap between dominant bacterial genera and extracellular enzyme activities (BG, NAG, LAP, CBH, ACP, POD, PPO). **(D)** RDA of fungal communities constrained by the same soil environmental indicators as in **(A)**. **(E)** Spearman correlation heatmap between dominant fungal genera and soil carbon/nutrient indicators. **(F)** Spearman correlation heatmap between dominant fungal genera and extracellular enzyme activities. Arrows in RDA plots represent environmental vectors; percentages on axes indicate the variance explained by each canonical axis. *: P < 0.05, **: P < 0.01, ***: P < 0.001.

Different straw incorporation regimes significantly altered the linkages between rhizosphere microbial communities and soil C, N, and P cycling processes. Notably, deep straw incorporation with accelerated decomposition (SD and SDR) showed the strongest correlations between microbial taxa, soil environmental factors, and enzyme activities at both the bacterial and fungal levels (**Figure 5**).

Redundancy analysis (RDA) revealed that the bacterial and fungal communities under SD and SDR were closely positively associated with multiple key soil environmental factors, including DOC, TDC, MBC, SOC, AN, and AP (**Figures 5A, D**). These results indicate that deep straw incorporation with a decomposition accelerator significantly strengthens the response of microbial communities to changes in carbon substrate availability and soil nutrient status. Correlation heatmaps further showed that the bacterial genera enriched under SD and SDR—including *Burkholderia-Caballeronia-Paraburkholderia*, *Sphingomonas*, and *Bacillus*—were significantly and positively correlated with SOC, MBC, DOC, and AP (*P* < 0.05; **Figure 5B**). This pattern reflects that deep straw incorporation with accelerated decomposition activates copiotrophic decomposer taxa, and intensifies the coupling of soil C and nutrient cycling. These enriched bacterial genera were also strongly positively correlated with the activities of C-, N-, and P-cycling extracellular enzymes, including BG, NAG, CBH, and ACP (**Figure 5C**). This indicates that deep straw incorporation enhances organic matter breakdown and nutrient release by stimulating the metabolic activity of these functional bacterial taxa.

At the fungal level, the beneficial taxa significantly enriched under SD and SDR—including *Trichoderma*, *Mortierella*, and *Saitozyma*—showed strong positive correlations with SOC, MBC, AP, and TP (**Figure 5E**), and were also positively correlated with the activities of C-, N-, and P-cycling enzymes (**Figure 5F**). These results suggest that deep straw incorporation promotes the development of fungal-mediated decomposition systems, and enhances the ecological competitiveness of beneficial fungal groups.

Collectively, the SD and SDR treatments established a highly efficient coupled system centered on the “microbe–enzyme–nutrient cycling” axis. This was achieved by increasing rhizosphere carbon availability, stimulating extracellular enzyme secretion, and reshaping the composition and functional potential of microbial communities. This system provides the core biological mechanism underlying the improvements in soil aggregate formation, nutrient retention, and ultimately peanut yield observed in this study.

### 3.8 Integrated regulatory pathways of straw incorporation on peanut yield and soil functions

**Figure 6:**
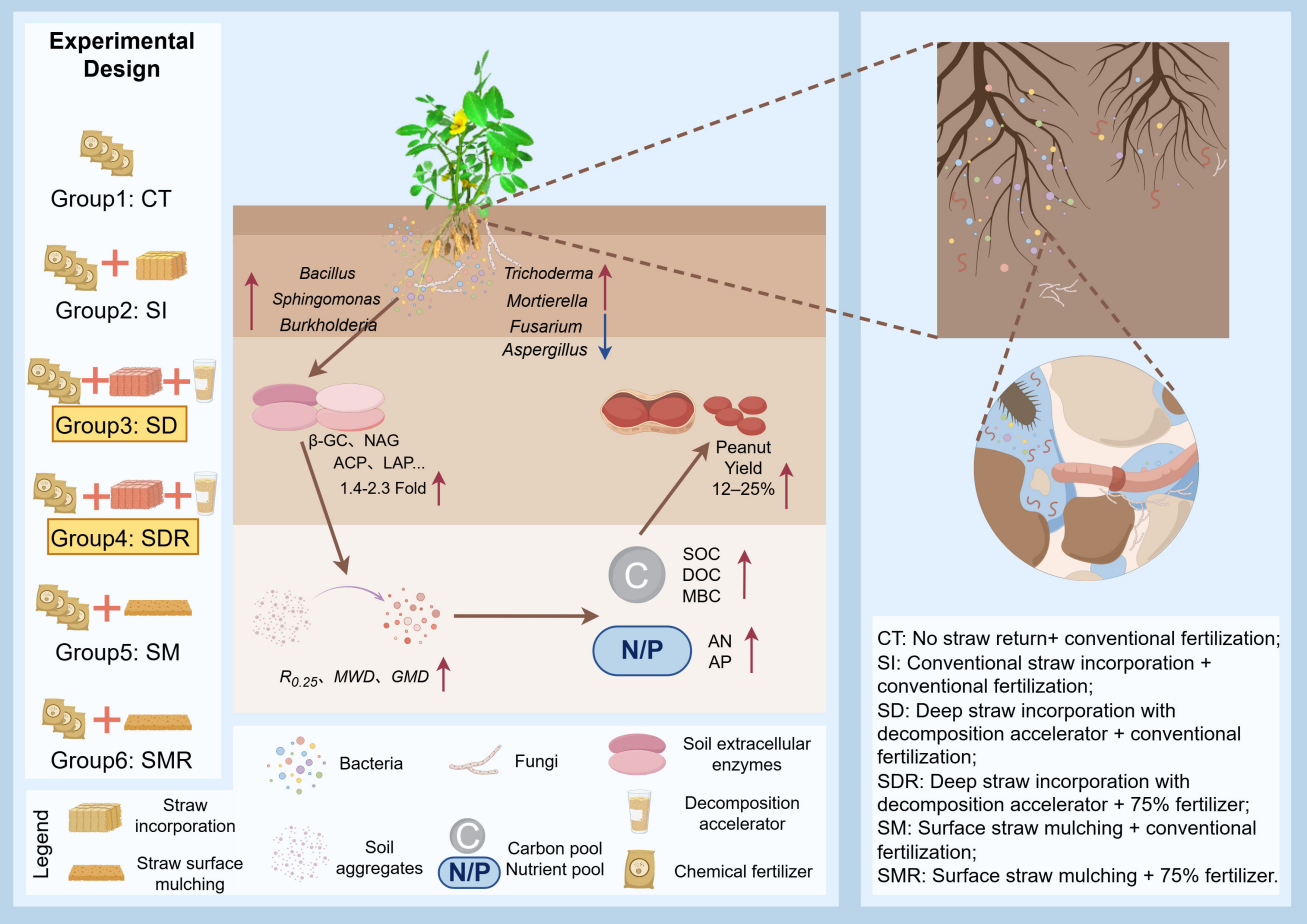
Schematic diagram illustrating the regulatory pathways of wheat straw incorporation regimes and fertilizer reduction on peanut yield and rhizosphere soil functions. The diagram includes the six experimental treatments (CT, SI, SD, SDR, SM, SMR) and their impacts on the “microbe–extracellular enzyme–soil aggregate–carbon/nutrient pool–peanut yield” cascade. Key components include beneficial microbial taxa, C/N/P-cycling and oxidative enzymes, soil aggregate stability indicators (*R_0_._25_*, MWD, GMD), rhizosphere carbon and nutrient pools, and peanut yield responses. Arrows indicate direct regulatory effects, with bold arrows highlighting the dominant regulatory pathways in the SD and SDR treatments. This schematic was created using the Figdraw platform.

To synthesize the integrated effects of wheat straw incorporation regimes and fertilizer reduction on peanut yield and rhizosphere soil functions, we constructed a schematic diagram illustrating the key regulatory pathways identified in this three-year field experiment (**Figure 6**). The schematic systematically presents the six experimental treatments and their divergent impacts on the “microbe–enzyme–aggregate–nutrient cycling–yield” cascade, consistent with the results presented in **Tables 1**, **2** and **Figures 1**–**5**.

Specifically, the SD and SDR treatments exhibited distinct advantages over all other treatments. These two treatments selectively enriched beneficial bacterial genera (*Bacillus*, *Sphingomonas*, *Burkholderia-Caballeronia-Paraburkholderia*) and fungal taxa (*Trichoderma*, *Mortierella*), which in turn significantly increased the activities of C/N/P-cycling extracellular enzymes (by 1.4–2.3-fold) and oxidative enzymes relative to CT. These enhanced enzymatic responses, coupled with the formation of soil aggregates mediated by microbial metabolites, significantly improved soil structural stability, as evidenced by the higher *R_0_._25_*, MWD, and GMD values (SD increased *R_0_._25_* by 26.4% and MWD by 98.3% compared with CT). Concomitantly, SD and SDR reshaped rhizosphere carbon pool dynamics (elevated SOC, DOC, and MBC) and enhanced soil nutrient availability (AN and AP), creating a favorable rhizosphere environment for peanut growth and nutrient uptake.

This integrated cascade of effects underpinned a 12–25% increase in three-year mean peanut yield under SD and SDR relative to CT. Critically, there was no statistically significant difference in yield between SD and SDR (*P* > 0.05), despite the 25% reduction in mineral fertilizer inputs in the SDR treatment. In contrast, the CT, SI, SM, and SMR treatments showed weaker enrichment of functional microbial taxa, lower extracellular enzyme activities, less improvement in soil aggregate stability, and relatively limited enhancement of soil carbon and nutrient pools, resulting in significantly lower peanut yields compared with SD and SDR. This schematic consolidates the core findings of the study, highlighting the central role of deep straw incorporation with a decomposition accelerator in coordinating rhizosphere microbial communities, enzyme activities, soil structure, and nutrient cycling to sustain high peanut yield under moderate fertilizer reduction.

## 4 Discussion

### 4.1 Effects of straw incorporation regimes on soil structure, extracellular enzyme activities, and nutrient retention

Our findings show that SD and SDR improved soil structure, increased extracellular enzyme activities, and enhanced soil nutrient retention. SD and SDR significantly increased *R_0.25_* as well as MWD and GMD compared to the CT (**Table 2**). While other straw-incorporation treatments (SI, SM, SMR) also showed improvements in some stability indicators relative to CT, the deep incorporation treatments consistently yielded the highest values. These improvements reflect the combined effects of organic binding agents released during straw decomposition and functional shifts in soil microbial communities. The decomposition accelerator introduced functional microbes (*Bacillus subtilis*, *Trichoderma harzianum*) whose cellulases and ligninases accelerate straw breakdown into dissolved organic carbon (DOC), supplying abundant energy for soil microbial metabolism. Concurrently, bacterial extracellular polysaccharides (EPS) intertwined with fungal hyphae to promote the conversion of microaggregates into macroaggregates (Meng et al., 2024; Liu et al., 2022). In our study, SD increased *R_0.25_* by 26.4% and MWD by 98.3% relative to CT, consistent with reports that straw incorporation enhances aggregate stability via microbially mediated binding (Zhang et al., 2026). Notably, SDR achieved aggregate stability comparable to SD despite a 25% fertilizer reduction (*P* > 0.05). This outcome may reflect nutrient release from straw decomposition that partly substitutes for mineral fertilizers. In addition, microorganisms can immobilize nutrients and secrete binding polymers, buffering potential aggregate breakdown under fertilizer reduction (Meng et al., 2024). Furthermore, macropore structures within large aggregates physically protect organic C from rapid decomposition, reinforcing an “aggregate–C sequestration” feedback. This aligns with the view that deep straw incorporation stabilizes soil C pools via aggregate-mediated physical protection (Zhou et al., 2023).

SD and SDR significantly elevated the activities of C-, N-, and P-cycling hydrolytic enzymes and oxidative enzymes (**Figure 1**). Elevated C-cycling enzymes–BG, BX, and CBH-suggest enhanced microbial energy acquisition via accelerated cellulose and hemicellulose degradation (He et al., 2025). Increased NAG, LAP, and ACP activities indicate greater microbial demand for N and P mineralization to balance C metabolism (Mori et al., 2021). Ecoenzymatic stoichiometry showed BG/(NAG+LAP) ratios of 1.2–1.5 under SD/SDR, significantly lower than CT (2.3). This suggests reduced microbial C limitation and enhanced N transformation under deep, accelerated decomposition (Zheng et al., 2022). The BG/(NAG+LAP) ratio reflects microbial resource allocation between C and N acquisition. A lower ratio under SD/SDR means microbes allocate less effort to C-degrading enzymes (BG) and relatively more to N-acquiring enzymes (NAG+LAP), indicating reduced C limitation (sufficient C substrates from straw decomposition) and enhanced N limitation for microbial metabolism. Furthermore, higher oxidase activities (PPO, POD) promote lignin humification, and the formed humic substances further strengthen aggregate binding (Qiao et al., 2024; Song et al., 2024b). Notably, enzyme activities under SDR did not differ from SD (*P* > 0.05), consistent with reports that straw incorporation offsets fertilizer reduction by enhancing microbial P cycling (Pu et al., 2023). Thus, under deep incorporation with decomposition acceleration, enhanced microbial nutrient cycling can substitute for part of the nutrient supply provided by mineral fertilizers.

SD and SDR significantly increased soil organic carbon (SOC), dissolved organic carbon (DOC), microbial biomass carbon (MBC), available nitrogen (AN), and particulate organic carbon (POC). This enhanced nutrient retention was driven by synergistic physical protection within soil aggregates and biological immobilization by microbial communities (**Figure 2**), with macroaggregate formation directly reducing nutrient leaching losses. Internal closed pores can adsorb and immobilize plant-available N and P, lowering the risk of nutrient migration (Li et al., 2025b). Here, SOC under SD increased by 32.6% versus CT, with ~45% of SOC contained in >0.25-mm macroaggregates. This agrees with the view that macroaggregates act as long-term reservoirs of soil organic C (Pan et al., 2025). Biologically, higher MBC indicates greater microbial nutrient sequestration. MBC under SD/SDR increased by 48.6%–55.3% relative to CT. Microorganisms can assimilate available nutrients into biomass N and P, preventing losses while slowly releasing them for plant uptake (Yang et al., 2024). Moreover, decomposition acceleration deepens N and P mineralization from straw. Released nutrients can bind to functional groups on soil colloids and aggregate surfaces, forming relatively stable nutrient pools. In this study, SDR increased AN and AP by 35.9% and 45.4%, respectively, versus CT, and did not differ from SD. This aligns with reports that straw incorporation enhances nutrient retention via combined physical–biological mechanisms, permitting ~20–30% fertilizer reduction without compromising supply (Wang et al., 2023). In legumes such as peanut, deep straw incorporation with decomposition acceleration can maintain or increase yield while lowering fertilizer intensity per unit yield and potential environmental costs (e.g., N leaching, N_2_O emissions). This approach supports dual goals of higher “green” yield and improved soil health (Yang et al., 2024).

### 4.2 Responses of rhizosphere microbial communities to straw incorporation regimes

Straw incorporation methods substantially shaped rhizosphere bacterial and fungal community structure and function. Deep incorporation with decomposition acceleration built an efficient microbe-driven system by selectively enriching functional taxa, strengthening interactions, and increasing functional redundancy (**Figures 3**, **4**). SD/SDR enriched bacterial genera such as *Bacillus*, *Sphingomonas*, *Burkholderia–Caballeronia–Paraburkholderia* and fungal genera such as *Trichoderma* and *Mortierella*. These enriched taxa are functionally linked to straw decomposition, nutrient transformation, and plant growth promotion. *Bacillus* spp. secrete cellulases and proteases, with their relative abundance positively correlated with C/N-cycling enzyme activities (*P* < 0.05) to accelerate straw organic matter degradation (He et al., 2025). *Sphingomonas*, which degrades lignin and produces humic-like binding compounds to support aggregate formation, was positively correlated with MBC and SOC (*P* < 0.05) (Gu et al., 2024). *Burkholderia* is known to solubilize P and increase soil available P. In our data, its abundance correlated positively with AP, MBC, and acid phosphatase (ACP) activity (*P* < 0.01), consistent with reports identifying *Burkholderia* as a core P-cycling taxon that mediates P transformation via enzyme secretion (Wei et al., 2024). For fungi, *Trichoderma* facilitates straw decomposition via enzymes and can suppress pathogens such as *Fusarium*. *Trichoderma* abundance was ~2.3-fold higher under SD/SDR than CT, whereas *Fusarium* decreased by ~65%. This is consistent with reports that *Trichoderma* achieves biocontrol via nutrient competition and antimicrobial secretion (Li et al., 2022). *Penicillium* mycelial networks can enhance aggregate stability and participate in lipid/protein decomposition, releasing plant-available N. In our study, *Penicillium* correlated positively with AN and DIC (*P* < 0.05), consistent with prior work (Zhu et al., 2025). Functional predictions indicated higher relative abundances of carbohydrate, amino-acid, and energy-metabolism pathways under SD/SDR than other treatments. These patterns further support enhanced metabolic activity of functional communities (Xie et al., 2022).

Bacterial co-occurrence networks identified *Bacillus*, *Sphingomonas*, and *Bradyrhizobium* as core nodes. High connectivity suggests pivotal roles in coordinating C–N cycling and nutrient transformations (**Figure 3H**), consistent with previous studies (Dixon et al., 2024). In fungal networks, *Trichoderma* and *Aspergillus* emerged as core nodes, indicating synergistic interactions that support straw decomposition and soil functions (**Figure 4H**). Deep straw incorporation with decomposition acceleration not only shifts taxa abundances but also optimizes fungal–bacterial synergy and niche partitioning. Bacteria mainly decompose labile components (e.g., starch, hemicellulose), releasing readily available nutrients. By contrast, fungi specialize in recalcitrant substrates (e.g., lignin, cellulose) and contribute to aggregate construction via mycelial networks. In our study, synergy between arbuscular mycorrhizal fungi (e.g., *Paraglomus*) and bacteria was strengthened. Mycorrhizal hyphae expanded nutrient uptake, while bacterial organic acids activated soil-bound P. This synergy enhanced peanut N and P uptake efficiency. These observations agree with reports that mycorrhizal–bacterial interactions improve crop nutrient-use efficiency (Ngosong et al., 2022; Wang et al., 2022).

It is important to note that the significant enrichment of *Bacillus* and *Trichoderma* in the SD and SDR treatments likely resulted from a combination of the direct colonization by the inoculated strains within the decomposition accelerator and the concurrent stimulation of indigenous populations. While the applied accelerator specifically contained *Bacillus subtilis* and *Trichoderma harzianum*, the 16S rRNA and ITS amplicon sequencing utilized in this study provides genus-level resolution and cannot definitively distinguish the introduced strains from native taxa. Nevertheless, the synergistic proliferation of these beneficial genera–whether introduced or indigenous–unmistakably contributed to accelerated straw degradation, pathogen suppression, and enhanced rhizosphere functionality.

### 4.3 Integrating microbial-enzymatic mechanisms into sustainable peanut production

This study elucidates a cascade mechanism whereby deep straw incorporation (Key Driver) enriches specific functional microbes, which in turn elevate extracellular enzyme activities (Key Process), leading to enhanced aggregate formation and nutrient cycling, and ultimately sustaining high crop yield under reduced fertilizer input. Three years of consecutive field data confirmed that SD and SDR treatments delivered stable, cumulative improvements in peanut yield, soil structure, and rhizosphere microbial function: peanut yield under SDR increased by 8.4% from 2022 to 2024, with soil MWD increasing by 12.3% over the same period, highlighting the long-term beneficial effects of this practice. Interannual comparisons revealed that the differences in soil enzyme activities and microbial diversity between SD and SDR were small in 2022, and narrowed further in the 2023 and 2024 growing seasons. This pattern likely reflects the continuous accumulation of soil organic C pools from repeated straw inputs over the three-year experiment. In turn, increased C availability may enable the soil microbial community to adapt to reduced fertilizer conditions and achieve greater functional stability over time. This is consistent with previous reports that long-term straw return enhances the functional stability of soil microbial communities via the accumulation of stable soil organic C pools. Moreover, the three-year dataset showed that the CV of peanut yield under SDR was only 3.2%, markedly lower than that under CT (7.8%). This indicates that the combination of deep straw incorporation, decomposition accelerator, and reduced fertilizer input enhances the resistance of crop productivity to environmental disturbances, which is critical for maintaining yield stability under climate variability and soil degradation stresses (He et al., 2025). Concurrently, the SDR treatment reduced mineral fertilizer input by 25%, while also lowering the risks of N and P leaching and greenhouse gas (GHG) emissions, increasing soil organic C sequestration, and reducing agricultural input costs. Together, these outcomes create a synergistic effect of “quality improvement, input reduction, and efficiency enhancement” for peanut production.

The ability of SDR to maintain yields equivalent to full-fertilized SD despite a 25% reduction in mineral fertilizer is driven by improved nutrient use efficiency (NUE) via plant-soil-microbe synergism. SD and SDR treatments increased plant nutrient use efficiency by 18.7% and 16.3%, respectively, compared with CT. This effect stems from sustained microbial mineralization of straw-derived nutrients, enhanced nutrient accessibility to peanut roots, and reduced leaching losses via physical protection within stable macroaggregates formed under deep straw incorporation. Simultaneously, the enriched fungal networks (e.g., *Trichoderma* and arbuscular mycorrhizal taxa like *Paraglomus*) effectively expanded the root foraging area. Consequently, this robust biological and physical buffering capacity within the modified rhizosphere successfully substituted for the reduced mineral fertilizer inputs, proving the viability of this low-input agronomic model.

Our results provide robust evidence for the efficacy of deep straw incorporation with a decomposition accelerator plus 25% fertilizer reduction in the Huang-Huai-Hai Plain, the core peanut-producing region of China. The wider regional applicability of this cultivation model will depend on local soil type, climatic conditions, and cropping systems. For soil texture, this model is well suited to loam and light clay soils, which provide the aeration and water retention conditions conducive to microbial activity and straw decomposition. In sandy soils, additional organic matter inputs are recommended to further enhance soil aggregate stability and reduce nutrient leaching risks (Yan et al., 2023). Climatically, the hot and rainy summer season in the Huang-Huai-Hai region matches the peak peanut growth period, and the decomposition accelerator can leverage the warm and humid conditions to accelerate straw breakdown. This is consistent with previous meta-analysis findings that straw return delivers more pronounced beneficial effects in regions with hot and humid climates (Hu et al., 2025). For cropping systems, this model can be extended to wheat-peanut and maize-peanut rotation systems, where straw incorporation supplies nutrients for the subsequent peanut crop and continuously improves soil fertility within the rotation cycle. The model in this study is based on full straw return at a rate of 7.5 t·ha^-^¹; where straw supply is limited, the magnitude of fertilizer reduction should be adjusted accordingly (e.g., 15%–20% reduction) to maintain soil nutrient balance (Li et al., 2024b). Furthermore, this deep incorporation plus reduced fertilizer model can be integrated with other conservation agricultural practices such as no-tillage, mulching, and biochar amendment to amplify its agronomic and environmental benefits. For example, pairing deep straw incorporation with no-tillage reduces soil disturbance, protects aggregate structure, and further enhances soil C sequestration. Combining this model with biochar amendment can improve soil water and nutrient retention capacity and prolong the functional benefits of straw-derived organic matter. Integrating rhizobial inoculation with this system can further strengthen biological N fixation and mitigate any potential yield risks associated with fertilizer reduction (Zou et al., 2024).

## 5 Conclusion

Our three-year randomized complete block field experiment, which investigated the effects of different wheat straw incorporation regimes and fertilizer reduction on peanut productivity and rhizosphere soil functions, demonstrated that deep straw incorporation with a decomposition accelerator (SD and SDR) significantly outperformed conventional straw incorporation, surface straw mulching, and the no-straw control. Relative to CT, SD and SDR increased the three-year mean peanut yield by 12%–25%, with no statistically significant yield difference between SD and SDR. This confirms that a 25% reduction in mineral fertilizer input can be achieved without compromising peanut yield when combined with deep straw incorporation and a decomposition accelerator. The superior yield performance of SD and SDR was linked to multiple coordinated improvements in soil quality: enhanced soil structural stability (e.g., the mean weight diameter (MWD) increased by 98.3% and 79.6% under SD and SDR, respectively, relative to CT), 1.4–2.6-fold increases in the activities of C-, N-, and P-cycling extracellular enzymes, and improved rhizosphere carbon pool dynamics (higher SOC, DOC, and MBC) and nutrient availability (AN and AP). Mechanistically, deep straw incorporation with a decomposition accelerator selectively enriched beneficial bacterial genera (e.g., *Bacillus*, *Sphingomonas*, *Burkholderia-Caballeronia-Paraburkholderia*) and fungal taxa (e.g., *Trichoderma*, *Mortierella*), which are core functional groups associated with straw decomposition, nutrient transformation, and plant growth promotion. These treatments also strengthened the complexity of microbial co-occurrence networks and enhanced the metabolic potential of microbial communities related to carbohydrate, amino acid, and energy metabolism. This integrated “microbe-enzyme-aggregate-nutrient cycling” cascade enhanced soil fertility and nutrient retention capacity, which fully offset the 25% fertilizer reduction in the SDR treatment. Collectively, our findings identify deep straw incorporation with a decomposition accelerator plus 25% fertilizer reduction as a practical, resource-efficient strategy for sustainable peanut production in the Huang-Huai-Hai region and similar agroecosystems. This practice balances high and stable peanut yield, long-term soil health improvement, and reduced environmental footprint of agricultural production. Future research should validate the economic and environmental benefits of this integrated practice across diverse soil types and climatic regions, and investigate the long-term (>5 years) dynamics of soil organic carbon sequestration and microbial network stability under sustained straw return with reduced fertilizer inputs.

## Data Availability

The raw 16S rRNA and ITS amplicon sequencing data presented in this study are deposited in the NCBI Sequence Read Archive (SRA) repository under accession number PRJNA1467539 and PRJNA1467562. All soil physicochemical property data, enzyme activity measurements, and aggregate stability data are available in the Supplementary Material associated with this article.
